# Compared values of presepsin (sCD14-ST) and procalcitonin as early markers of outcome in severe sepsis and septic shock: a preliminary report from the Albumin Italian Outcome Sepsis (ALBIOS) study

**DOI:** 10.1186/cc11973

**Published:** 2013-03-19

**Authors:** P Caironi, S Masson, E Spanuth, R Thomae, R Fumagalli, A Pesenti, M Romero, G Tognoni, R Latini, L Gattinoni

**Affiliations:** 1 Fondazione IRCCS Ca' Granda - Ospedale Maggiore Policlinico, Università degli Studi di Milano, Milan, Italy; 2 Istituto di Ricerche Farmacologiche Mario Negri, Milan, Italy; 3 Diagnostic Engineering & Research GmbH, Heildelberg, Germany; 4 Mitsubishi Chemical Europe GmbH, Munich, Germany; 5 Ospedale San Gerardo, Monza, Italy; 6 Consorzio Mario Negri Sud, Santa Maria Imbaro, Italy

## Introduction

Sepsis results from complex interactions between infecting microorganisms and host responses, often leading to multiple organ failures and death. Over the years, its treatment has been standardized in early goal-oriented therapies, which may benefit from circulating biomarkers for early risk stratification. We aimed to evaluate the prognostic value of presepsin (sCD14-ST), a novel marker of bacterial infection.

## Methods

We performed a nested case-control study from the randomized controlled Albumin Italian Outcome Sepsis (ALBIOS) trial, enrolling patients with severe sepsis or septic shock from 100 ICUs in Italy. Fifty survivors and 50 nonsurvivors at ICU discharge were selected, matched for age, sex, center and time of enrollment after inclusion criteria were present. EDTA-plasma samples were collected at days 1, 2 and 7 after enrolment for presepsin (immune-chemiluminescence assay PATHFAST Presepsin, URL 320 pg/ml, CV 5%; Mitsubishi Chemicals) and procalcitonin assay (PCT, Elecsys BRAHMS Cobas^® ^PCT, URL 0.046 ng/ml, CV 8.8%; Roche Diagnostics).

## Results

Clinical characteristics were similar between the two groups, except for a worse SOFA score at day 1 in decedents. Presepsin at day 1 was significantly higher in decedents (2,268 (1,145 to 4,305) pg/ ml, median (Q1 to Q3)) than in survivors (1,184 (855 to 2,158) pg/ml, *P *= 0.001), while PCT did not differ (18.5 (3.3 to 45.7) vs. 10.8 (2.6 to 46.4) ng/ml, *P *= 0.31). Presepsin decreased over time in survivors, but remained elevated in decedents (974 (674 to 1,927) vs. 2,551 (1,438 to 5,624) pg/ml at day 7, *P *= 0.02 for time-survival interaction); PCT decreased similarly in the two groups (*P *= 0.19). Patients with early elevated presepsin had worse SOFA score, higher number of MOFs, hemodynamic instability (lower mean arterial pressure at baseline and after 6 hours), and mortality rate at 90 days (75% vs. 42%, log-rank *P *< 0.001). The association between presepsin and outcome was more marked in patients with late enrollment (6 to 24 hours), and in septic shock. Early presepsin had better prognostic accuracy than PCT (AUROC 0.69 vs. 0.56, *P *= 0.07), and improved discrimination over SOFA score, especially in septic shock.

## Conclusion

Early presepsin measurements may provide important prognostic information in patients with severe sepsis or septic shock, and may be of crucial importance for early risk stratification.

